# Identifying patients at risk for myasthenic crisis with hemogram and inflammation-related laboratory parameters – a pilot study

**DOI:** 10.3389/fneur.2024.1297997

**Published:** 2024-02-26

**Authors:** Anne Mehnert, Sivan Bershan, Jil Kollmus-Heege, Lea Gerischer, Meret Luise Herdick, Sarah Hoffmann, Sophie Lehnerer, Franziska Scheibe, Frauke Stascheit, Maike Stein, Alastair M. Buchan, Andreas Meisel, Annette Aigner, Philipp Mergenthaler

**Affiliations:** ^1^Charité - Universitätsmedizin Berlin, Department of Neurology with Experimental Neurology, Berlin, Germany; ^2^Charité - Universitätsmedizin Berlin, Center for Stroke Research Berlin, Berlin, Germany; ^3^Charité - Universitätsmedizin Berlin, Institute of Biometry and Clinical Epidemiology, Berlin, Germany; ^4^Charité - Universitätsmedizin Berlin, Neuroscience Clinical Research Center, Berlin, Germany; ^5^Berlin Institute of Health at Charité, Digital Health Center, Berlin, Germany; ^6^Radcliffe Department of Medicine, University of Oxford, Oxford, United Kingdom

**Keywords:** myasthenia gravis, myasthenia, myasthenic crisis, hemogram, laboratory parameters, inflammation, risk prediction

## Abstract

**Background:**

Myasthenia gravis (MG) is a rare autoimmune disease characterized by fatigable weakness of the voluntary muscles and can exacerbate to life-threatening myasthenic crisis (MC), requiring intensive care treatment. Routine laboratory parameters are a cost-effective and widely available method for estimating the clinical outcomes of several diseases, but so far, such parameters have not been established to detect disease progression in MG.

**Methods:**

We conducted a retrospective analysis of selected laboratory parameters related to inflammation and hemogram for MG patients with MC compared to MG patients without MC. To identify potential risk factors for MC, we applied time-varying Cox regression for time to MC and, as a sensitivity analysis, generalized estimating equations logistic regression for the occurrence of MC at the next patient visit.

**Results:**

15 of the 58 examined MG patients suffered at least one MC. There was no notable difference in the occurrence of MC by antibody status or sex. Both regression models showed that higher counts of basophils (per 0.01 unit increase: HR = 1.32, 95% CI = 1.02–1.70), neutrophils (per 1 unit increase: HR = 1.40, 95% CI = 1.14–1.72), potentially leukocytes (per 1 unit increase: HR = 1.15, 95% CI = 0.99–1.34), and platelets (per 100 units increase: HR = 1.54, 95% CI = 0.99–2.38) may indicate increased risk for a myasthenic crisis.

**Conclusion:**

This pilot study provides proof of the concept that increased counts of basophils, neutrophils, leukocytes, and platelets may be associated with a higher risk of developing MC in patients with MG.

## Introduction

Myasthenia gravis (MG) is a rare autoimmune disease caused by an antibody-mediated disturbance of signal transduction at the neuromuscular endplate. The main symptoms are fatigable weakness of the voluntary muscles, worsening with exertion, and fatigue (1). In 70–80% of all patients, MG is caused by pathogenic autoantibodies directed against the acetylcholine receptor (AChR) at the neuromuscular junction (2–4). The loss of functional AChR leads to a reduced amplitude of the endplate potential and, thus, impeded neurotransmission at the neuromuscular endplate (5, 6). MG manifests at the extraocular muscles, leading to ptosis or double vision, and by generalized or bulbar weakness affecting limb muscles or oropharyngeal muscles at manifestation or as the disease progresses.

Critical exacerbation of these symptoms can lead to life-threatening myasthenic crisis (MC), which often requires intensive care treatment with non-invasive or even invasive ventilation and invasive therapy (7). An MC often occurs within the first few years of the disease and can be the first manifestation of MG (8). The lifetime prevalence of MC is 15–20% for patients with MG (9, 10). MC-associated mortality is commonly reported between 5 and 12% (10–13), but mortality up to 22% has also been reported (14, 15). Furthermore, it is established that antibody status or clinical treatment protocols are associated with outcomes after MC (10, 13, 16, 17).

Although it is known that certain drugs, inadequate treatment, surgery, infection, sepsis, and pregnancy can trigger MC (9, 18, 19), the prediction of severe exacerbation of MG or ultimately MC based on laboratory parameters is currently not possible. To this end, so far only a few studies have investigated the relationship between hemogram or inflammation-related laboratory parameters and the disease progression of MG (20–22).

Thus, we hypothesized that certain laboratory parameters could be used to evaluate disease activity in MG even before clinically obvious exacerbations and to identify patients at risk of progressing to MC. We studied highly granular laboratory parameters related to inflammation and hemogram in patients suffering from MC prior to the event, compared to MG without MC to investigate if changes in these parameters could be indicative of the development of MC. This retrospective case–control study with a small number of subjects serves as a pilot study, whose concept and results could later be validated in a larger cohort.

## Materials and methods

### Standard protocol approvals, registrations, and patient consent

This study was approved by the ethics committee at Charité – Universitätsmedizin Berlin (no. EA4/068/22). Data were collected retrospectively. Due to the retrospective nature, individual patient consent was not obtained in accordance with ethical approval and state and national laws. This manuscript has been posted as a preprint on medRxiv prior to submission to this journal (23).

### Study design and patient selection

For this study, we evaluated clinical data from 58 MG patients treated at the integrated Myasthenia Center of the Department of Neurology at Charité – Universitätsmedizin Berlin. It is certified by the German Myasthenia Gravis Society and employs standardized workflows for patient management. The diagnosis of MG was established based on antibody studies, repetitive nerve stimulation, or clinical assessment. MC was defined as the exacerbation of myasthenic symptoms with bulbar or general weakness requiring mechanical ventilation. First, we selected 15 patients who were treated for MC at least once and for whom sufficiently complete medical data were available from all MC patients at our center between 2006 and 2016. MC patients were intended to be matched in a 1:3 ratio with MG patients treated at our center without recorded MC until 2018. Although data for MC patients were available after 2016, they were not included in the analysis to avoid hindsight bias. Matching was based on the criteria sex, age ± 5 years, antibody status (AChR antibodies or negative for AChR, MuSK, LRP4), thymectomy (yes/no), and thymus pathology (thymoma, thymus hyperplasia, and unremarkable). Due to insufficient matching partners with applicable matching criteria, one MC patient could only be matched with one control patient. The final cohort consisted of 58 subjects (15 MC, 43 non-MC patients).

In this study, we focused on the analysis of the following laboratory parameters: hemoglobin, hematocrit, mean corpuscular hemoglobin (MCH), mean corpuscular hemoglobin concentration (MCHC), white blood cell count, white blood cell differential count (basophils, eosinophils, monocytes, lymphocytes, neutrophils, and granulocytes), platelet count, and C-reactive protein (CRP). Selected laboratory data were obtained through the Berlin Institute of Health at Charité Health Data Platform (HDP), which hosts up-to-date retrospective data on the hospital management system. For this pilot study, we retrieved all available data for the curated list of laboratory parameters of the selected patients over the entire observation period from 2006 to 2018. For the analysis, we only considered data obtained prior to the occurrence of an MC.

### Statistical analysis

We descriptively display all patient characteristics used for matching, separately for cases and controls. Categorical variables are presented as absolute and relative frequencies. To summarize the laboratory parameters, we display the first measurement per patient (baseline) as well as the median value per patient measured before the beginning of the first MC with the median and interquartile range (IQR). Kaplan–Meier curves display the time to first MC stratified by sex (male or female) and antibody status (AChR positive or negative).

We used an Anderson–Gill model, a time-varying Cox proportional-hazards regression, with time to MC as the outcome. To account for the dependency in the data, we used robust standard errors. Time was modeled since the first observation, and patients without any further crises were censored at the time of the database excerpt. This model assumes that the risk of experiencing an MC remains the same, irrespective of whether previous events occurred or not. This means that after an MC has occurred, a subject is treated the same way as a subject who has not experienced an MC. As sensitivity analysis, we also performed a generalized estimating equations (GEE) logistic regression model, which explains the binary outcome of a potential current crisis with the laboratory parameter measured at the prior visit.

Because of the initial matching, we do not adjust for age and sex in any of the models. Due to the limited number of observed events, all models ran for each laboratory parameter separately (univariable models). Using complete-case analyses, these models are therefore based on a different number of observations, due to the clinical practice of not measuring all laboratory parameters at every time point. Based on these models, we derived hazard ratio (HR) and odds ratio (OR) estimates along with 95% confidence intervals (CI). All analyses were performed using R [R Project for Statistical Computing (24)], as well as additional R packages for data handling and analysis (25–27).

## Results

### Demographics and clinical characteristics

This pilot study included 58 patients (30 female, 28 male), of whom 15 (26%) suffered from one or more MC (cases) and 43 never had an MC (controls) within the observation period. In the case group, 11 patients suffered one MC, 2 patients suffered two MCs, and 2 patients suffered three MCs. In total, there were 21 MC events (Table 1). Both baseline (i.e., first-ever recorded) and median values for all available measurements prior to MC were similar for all laboratory parameters in both groups. CRP differed in cases and controls (Table 2). The frequency of measurements per person and laboratory parameter varied. For the controls, the median number of observations was 8 (IQR: 3–26, min = 1, max = 714), and for cases 42 (IQR: 18–67.5, min = 1, max = 101). The median number of measurements for the complete blood count with differential was 8 (IQR: 2–19.5, min: 1, max: 56) and 15 (IQR: 4–33, min: 1, max: 158) without differential. For CRP, it was 6 (IQR: 2–15.5, min: 1, max: 93). The median time between two measurements was 3 days (IQR: 2–35, min: 1, max: 3135). Stratified by the outcome, for the 1944 observations where no MC occurred in the subsequent visit, the median time to next visit (i.e., time to next measurement) was 3 days (IQR: 2–34, min: 1, max: 3135) and 39 days (IQR: 19.2–83.8, min: 1, max: 2196) for the 20 observations where an MC occurred. In the MC group, bulbar symptoms preceded the recorded MC in 17 of 21 events (81.0%). As there is no direct comparison to this measure for the control group, we rely on MGFA classification (i.e., the worst ever recorded MGFA) to categorize patients into groups presenting with mainly bulbar or generalized symptoms. Control patients presented with bulbar symptoms in 29 of 43 cases (67.4%) and generalized symptoms in 10 of 43 cases (23.3%). In four cases (9.3%), it was not possible to unequivocally determine the MGFA category.

**Table 1:** Cohort demographics and clinical characteristics.

	Myasthenic crisis (*n* = 15)	No myasthenic crisis (*n* = 43)
Sex		
Male, n (%)	7 (46.7)	21 (48.8)
Female, n (%)	8 (53.3)	22 (51.2)
Age		
Age at diagnosis, years, mean (SD)	56 (16.5)	51 (17.2)
Early onset MG, n (%)	6 (40.0%)	16 (37.2%)
Late onset MG, n (%)	9 (60.0%)	27 (62.8%)
Number of MC per patient		
0	-	43
1	11 (73.3%)	-
2	2 (13.3%)	-
3	2 (13.3%)	-
AChR antibodies		
Positive	10 (66.7%)*	28 (65.1%)
Negative for AChR, MuSK, LRP4	5 (33.3%)	15 (34.9%)
Thymectomy		
No, n (%)	5 (33.3%)	15 (34.9%)
Yes, n (%)	10 (66.7%)	28 (65.1%)
Thymus pathology		
Thymoma, n (%)	5 (50.0%)	14 (50.0%)
Hyperplasia, n (%)	2 (20.0%)	5 (18.0%)
Unremarkable, n (%)	3 (30.0%)	8 (29.0%)
Unknown, n (%)	–	1 (4.0%)

*One MC patient was found positive for AChR and MuSK. As there were no appropriate AChR/MuSK-double positive controls, she was considered in the AChR+ group.

**Table 2:** Hemogram and inflammation-related laboratory parameter measurements of patients.

	Mysthenic crisis (*n* = 15)	No myasthenic crisis (*n* = 43)
Basophils/nl (baseline)		
Median (IQR)	0.03 (0.02, 0.05)	0.03 (0.02, 0.05)
Missing	1 (6.7%)	1 (2.3%)
Basophils/nl (median)		
Median (IQR)	0.03 (0.02, 0.05)	0.04 (0.02, 0.05)
Missing	1 (6.7%)	1 (2.3%)
C-reactive protein in mg/l (baseline)		
Median (IQR)	7.10 (4.05, 14.93)	3.50 (1.37, 8.07)
Missing	–	19 (44.2%)
C-reactive protein in mg/l (median)		
Median (IQR)	12.00 (4.20, 34.42)	4.35 (1.68, 8.02)
Missing	–	19 (44.2%)
Eosinophils/nl (baseline)		
Median (IQR)	0.06 (0.03, 0.10)	0.08 (0.03, 0.14)
Missing	3 (20.0%)	3 (7.0%)
Eosinophils/nl (median)		
Median (IQR)	0.07 (0.06, 0.12)	0.08 (0.06, 0.14)
Missing	3 (20.0%)	3 (7.0%)
Hematocrit in l/l (baseline)		
Median (IQR)	0.43 (0.40, 0.46)	0.42 (0.40, 0.44)
Hematocrit in l/l (median)		
Median (IQR)	0.41 (0.37, 0.43)	0.42 (0.38, 0.43)
Hemoglobin in g/dl (baseline)		
Median (IQR)	14.20 (13.55, 15.65)	14.10 (13.40, 14.65)
Hemoglobin in g/dl (median)		
Median (IQR)	13.40 (12.15, 14.55)	13.80 (12.85, 14.60)
Leukocytes/nl (baseline)		
Median (IQR)	9.20 (7.28, 13.19)	8.20 (6.06, 10.28)
Leukocytes/nl (median)		
Median (IQR)	8.11 (6.99, 10.25)	7.60 (6.65, 8.96)
Lymphocytes/nl (baseline)		
Median (IQR)	1.57 (1.08, 1.64)	1.52 (1.05, 1.88)
Missing	1 (6.7%)	1 (2.3%)
Lymphocytes/nl (median)		
Median (IQR)	1.30 (0.88, 1.80)	1.33 (1.04, 1.71)
Missing	1 (6.7%)	1 (2.3%)
Mean corpuscular hemoglobin in pg (baseline)		
Median (IQR)	30.80 (29.05, 32.00)	30.70 (29.85, 31.75)
Mean corpuscular hemoglobin in pg (median)		
Median (IQR)	30.70 (29.45, 31.60)	30.60 (29.35, 31.80)
Mean corpuscular hemoglobin concentration in g/dl (baseline)		
Median (IQR)	33.30 (33.05, 34.35)	33.90 (33.30, 34.50)
Mean corpuscular hemoglobin concentration in g/dl (median)		
Median (IQR)	32.80 (32.60, 33.55)	33.60 (32.75, 34.33)
Monocytes absolute/nl (baseline)		
Median (IQR)	0.59 (0.25, 0.90)	0.51 (0.42, 0.66)
Missing	1 (6.7%)	1 (2.3%)
Monocytes absolute/nl (median)		
Median (IQR)	0.63 (0.47, 0.81)	0.56 (0.49, 0.69)
Missing	1 (6.7%)	1 (2.3%)
Neutrophils/nl (baseline)		
Median (IQR)	6.98 (4.88, 10.75)	5.43 (3.81, 8.01)
Missing	1 (6.7%)	1 (2.3%)
Neutrophils/nl (median)		
Median (IQR)	6.40 (5.41, 7.89)	5.35 (4.25, 6.31)
Missing	1 (6.7%)	1 (2.3%)
Platelets/nl (baseline)		
Median (IQR)	268.00 (220.50, 324.00)	246.00 (206.50, 283.50)
Platelets/nl (median)		
Median (IQR)	260.00 (214.50, 297.00)	249.50 (210.25, 279.50)
Immature granulocytes/nl (baseline)		
Median (IQR)	0.04 (0.02, 0.06)	0.03 (0.01, 0.04)
Missing	1 (6.7%)	1 (2.3%)
Immature granulocytes/nl (median)		
Median (IQR)	0.04 (0.03, 0.07)	0.03 (0.02, 0.05)
Missing	1 (6.7%)	1 (2.3%)

For each laboratory parameter, the baseline (first measurement recorded per patient) and the median of all measurements recorded within the observation period before the beginning of the first MC are shown. Missing data are indicated where observed.

### Time to first myasthenic crisis stratified by sex and antibody status

**Figure 1:**
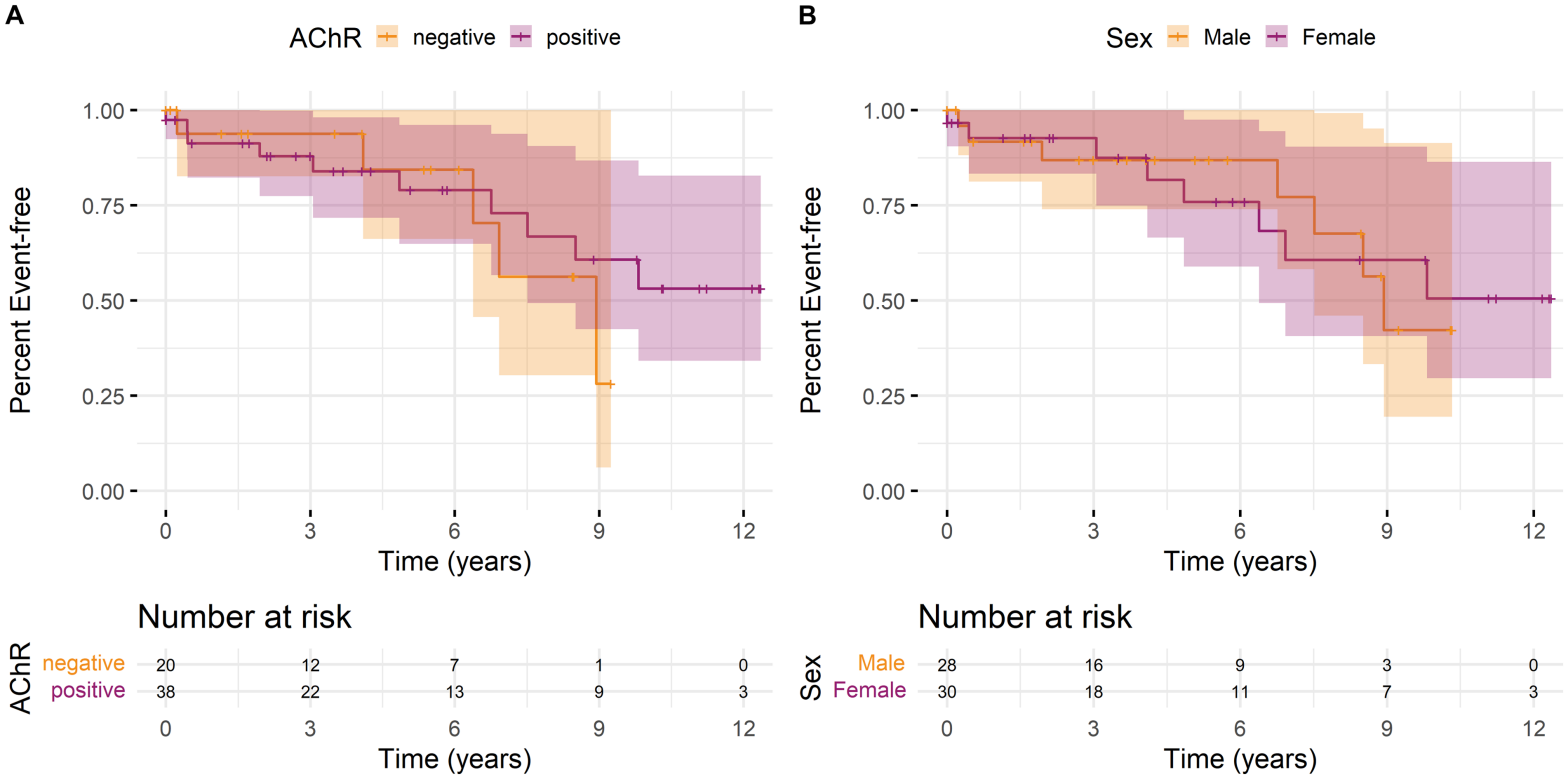
Kaplan–Meier curves with 95% confidence intervals (shaded areas) for time to first myasthenic crisis since the first recorded laboratory parameter stratified by **(A)** antibody status or **(B)** sex over the observation period. An event is defined as the first MC of a patient. The individual lines show the stratified survival curves, and the shaded areas present 95% confidence interval. **(A)** Orange lines represent the data for AChR antibody-negative patients and purple lines for AChR antibody-positive patients. **(B)** Orange lines represent the data for male and purple for female patients. Vertical bars indicate the censorship of an observation at that point. Additionally, the number of individuals at risk for a first MC at regular time points is shown below the curves.

We calculated Kaplan–Meier curves for time to the first MC since the first recorded laboratory parameter, stratified by AChR antibody status and sex. Overall, there was neither a significant difference in the occurrence of MC depending on antibody status (Figure 1A) nor between women and men (Figure 1B).

### Laboratory parameters associated with myasthenic crisis

Both statistical models we applied make use of the previous measurement to explain the occurrence of an event (MC or no MC). Laboratory parameter measurements from 1964 observations were used to explain 20 events with subsequent MC (one MC did not have sufficiently complete data to be included) and 1944 observations without subsequent MC.

**Figure 2:**
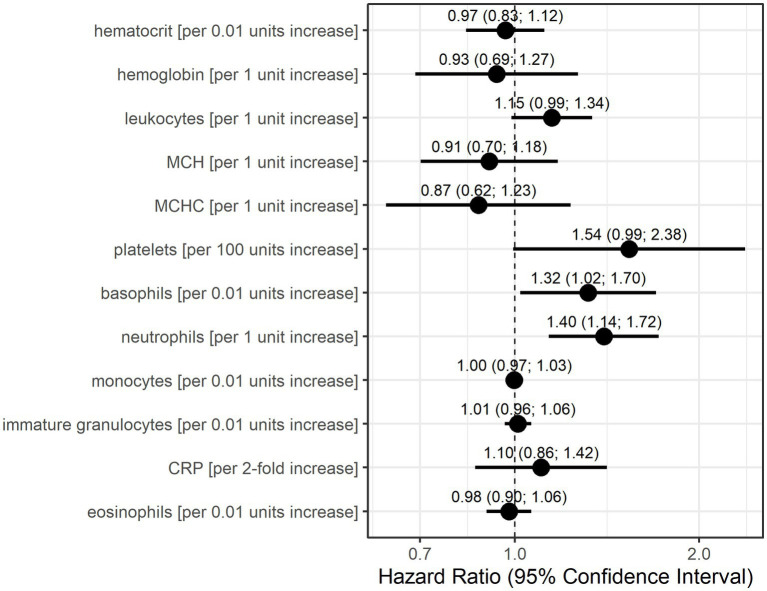
Hazard ratio estimates with 95% confidence intervals based on Anderson-Gill models for each laboratory parameter for the occurrence of myasthenic crisis. Dots indicate hazard ratio estimates for each laboratory parameter, and horizontal bars indicate 95% confidence intervals (in parentheses). Units for each laboratory parameter are shown in Table 2.

Univariable Anderson–Gill models showed that basophils, neutrophils, and potentially leukocytes and platelets indicate increase hazards for a myasthenic crisis (Figure 2). Without adjustment for other parameters, an increase of basophils by 0.01 units increased the risk of an MC 1.32-fold (95% CI: 1.02–1.70) and a 1 unit increase in neutrophils 1.4-fold (95% CI: 1.14–1.72). Furthermore, every unit increase in leukocytes increased the hazard for MC 1.15-fold (95% CI: 0.99–1.34), and an increase of 100 units in platelets 1.54-fold (95% CI: 0.99–2.38).

**Figure 3:**
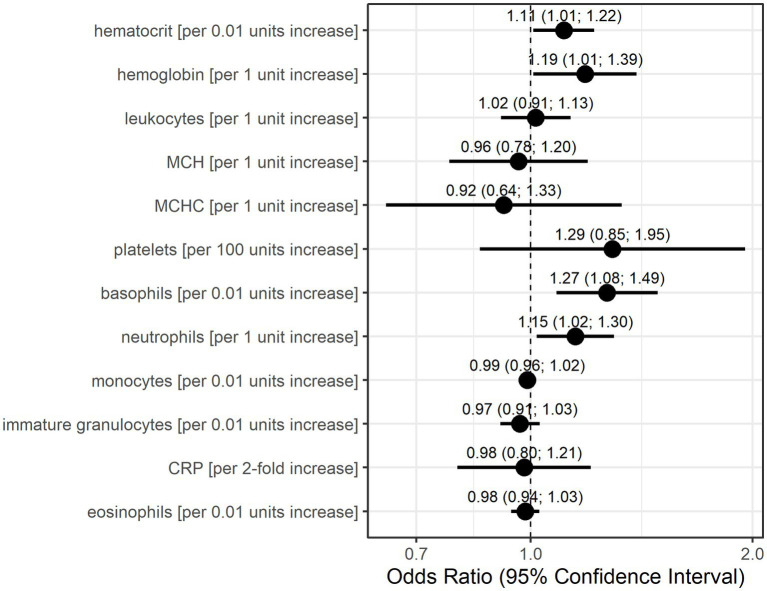
Odds ratio estimates and 95% confidence intervals based on generalized estimating equations and logistic regression for each individual laboratory parameter for the occurrence of myasthenic crisis at the subsequent visit. Dots indicate odds ratio estimates for each laboratory parameter, and horizontal bars indicate 95% confidence intervals (in parentheses). Estimates were derived from the generalized estimating equations (GEE) logistic regression. Units for each laboratory parameter are shown in Table 2.

The GEE logistic regression models conducted as sensitivity analyses with the occurrence of an MC in the subsequent patient visit as the outcome also identified basophils, neutrophils, and platelets as potentially relevant laboratory parameters (Figure 3). The odds for MC in the subsequent visit were 1.27-fold (95% CI: 1.08–1.49) per 0.01 unit increase in basophils and 1.15-fold (95% CI: 1.02–1.30) per 1 unit increase in neutrophils. A 100-unit increase in platelets increased the odds for an event 1.29-fold (95% CI: 0.85–1.95), although this association was calculated with low precision. Additionally, higher values in hematocrit (per 0.01 units) and hemoglobin (per 1 unit) resulted in higher odds for a subsequent MC (OR = 1.11, 95% CI: 1.01–1.22, OR = 1.19, 95% CI: 1.01–1.39, respectively).

Both statistical models consider data only before the occurrence of an MC but differently account for time. The Anderson–Gill model is a time-varying Cox regression model and, as such, explores the relationship between the time to the occurrence of an event and the explanatory variables. The dependent variable here is the hazard function at a given time t. Therefore, the model is dependent on time, as the hazard of an MC occuring changes with time. The GEE logistic regression estimates the odds for an event (here the occurrence of an MC at the next visit) based on the explanatory variables in the model. Time is only taken into account by the sequence of measurements and the occurrence of an MC at the next visit. It is interesting to note that in 10 of 21 events (47.6%), patient records showed signs of (bacterial) infection prior to the respective MC. It is also noteworthy that infections prior to MC seemed to be more prevalent in male patients (5 of 8 events, 62.5%) than in female patients (5 of 13 events, 38.5%). However, CRP was not identified with increased risk for MC by either statistical model. Based on the results of these models, we conclude that increased values of basophils, neutrophils, and, with lower confidence, also leukocytes and platelets are associated with an increased risk of developing an MC.

## Discussion

In this pilot study, we investigated whether routine laboratory data could be used to anticipate the occurrence of MC during the disease course in MG patients. Based on two statistical models with distinct assumptions, we found that from our pre-selected set of laboratory parameters, higher basophils, neutrophils, leukocytes, and platelets measured before an event (i.e., MC) were associated with a higher risk of developing an MC. Although baseline and median CRP as the most obvious laboratory markers of infection (procalcitonin was not determined in any of the cases reported here) before MC were elevated in the MC group compared to the control group, it was not identified as a risk to develop an MC by either statistical model.

Several risk factors for MC have previously been identified from retrospective analyses: infections (15, 19, 28), drugs (28), corticosteroid treatment (29, 30), older age (31), thymoma (15), bulbar symptoms (15, 32), high disease severity (15, 31), male sex (15, 30), and the presence of additional autoimmune diseases (31, 32). Another risk factor for MC is surgery (15, 33), including thymectomy. Although some attempts to establish risk scores for the occurrence of postoperative myasthenic crisis have been made, the identified risk factors (bulbar symptoms, disease severity, decreased vital capacity, and thymoma) generally align with established common risk factors for MC (33–35). Due to the high mortality rate of MC of 5–12% (10, 13, 16, 18), which can be stratified by AChR (10) or MuSK (16) antibodies, and triple-seronegative patients (13), there is a significant need to establish risk scores or identify parameters that can be used to predict the occurrence of MC, and thus aid early intervention.

Our study provides initial indications that routine laboratory parameters assessed before the onset of MC could be used as risk predictors for MC occurrence and facilitate early interventions (e.g., treatment with immunoglobulins or plasma exchange), possibly preventing MC and mitigating the associated morbidity and mortality. To this end, some studies have investigated the prediction of in-hospital mortality in MC based on selected laboratory parameters (22, 36). A recent study derived a predictive score for in-hospital mortality of MC using the Myasthenia Gravis Foundation of America (MGFA) score at the onset of the MC, septic shock, and cardiac arrest (36). In addition, this study suggested that low serum albumin, low hemoglobin, and a high leukocyte count might be associated with a higher mortality in MC (36). The latter may corroborate our findings that increased leukocyte counts may be associated with an increased risk for an ensuing MC. Further studies described a possible association between infections (37) and signs of inflammation (leukocytosis) (22) and an increased risk of developing MC. Furthermore, a hemogram could provide clues to the course of the disease, as hematological changes have been identified as prognostic factors of mortality for several critical illnesses (22), e.g., endocarditis (38), acute kidney injury (39), and acute myocardial infarction (40, 41). Extreme leukocytosis and anemia have been described as important risk factors for increased mortality in MC (22). Similarly, elevated neutrophil-to-lymphocyte ratios have been reported to be a potential risk factor for indicating the disease severity of MG in children (20) and adults (21). It is interesting that, among others, we identified basophilia as a potential indicator for risk of MC. Classically, basophilia is seen in hypersensitivity reactions of the immediate type (type 1) (42). Basophils are thought to play a role in host defense against parasites (43). Consequently, associations of basophilia with chronic inflammation and autoimmunity have been described (42, 43). As such, our data might open novel opportunities to study biomarkers of disease activity in MG. Together with real-world routine clinical data, inexpensive laboratory studies could allow risk classification for MC that goes beyond known risk factors for MC, such as infection, as exemplified by a recent study that used explainable machine learning to classify the risk for MC based on these parameters (44).

There are several limitations to our study. The dataset, with 58 patients in total, is small. However, the dataset includes 21 MC events and several sequential laboratory measurements per patient since the laboratory parameters were measured frequently. This leads to an uneven distribution of measurements between cases and controls. It is possible that the elevated CRP in the MC group was not identified as a risk factor for MC because of the small size of our cohort. Approximately half of the MCs recorded here showed preceding infections, which is a known risk factor for MC. It is also possible that subgroups (e.g., males) could be more prone to developing MC after infection. However, our study was not designed to address such questions. Furthermore, there is the potential for selection bias due to the retrospective and monocentric design and hand-selection of cases and controls for this pilot study. This study did not consider further clinical data, such as information on infection or co-medication, which are known risk factors for the clinical worsening of MG. Similarly, steroids or steroid-sparing immunosuppression are standard medications in MG patients known to affect blood counts but were not considered confounders. We used complete-case analyses based on a different number of observations, as a result of clinical practice not to measure all laboratory parameters at every time point. This leads to a different number of measurements per parameter per patient, and thus they could only be considered as univariate parameters in the models.

In conclusion, this study indicates that increased basophils, neutrophils, leukocytes, and platelets may be associated with an increased risk for the occurrence of MC in MG patients. The results of this pilot study suggest that it is possible to identify predictors for MC risk based on routine laboratory data. Together with other medical data (44), routine blood biomarkers could serve to develop a risk prediction score to tailor individualized treatment decisions at the point of care. However, larger prospective studies beyond the proof of concept stage are necessary to verify our results.

## Data availability statement

The datasets presented in this article are not readily available because ethical approval currently does not permit sharing of raw data. Approval will be sought by the corresponding author upon reasonable request with scientific rationale and sound methodology. Requests for data sharing will be managed in accordance with data access and sharing policies of Charité – Universitätsmedizin Berlin. Requests to access the datasets should be directed to the corresponding author.

## Ethics statement

The studies involving humans were approved by the Ethics Committee of Charité – Universitätsmedizin Berlin. The studies were conducted in accordance with the local legislation and institutional requirements. Written informed consent for participation was not required from the participants or the participants’ legal guardians/next of kin because data were collected retrospectively. Due to the retrospective nature, individual patient consent was not obtained, in accordance with the ethics approval, and state and national laws.

## Author contributions

AMeh: Data curation, Formal analysis, Investigation, Writing – original draft. SB: Formal analysis, Investigation, Writing – review & editing. JK-H: Investigation, Writing – review & editing. LG: Writing – review & editing. MH: Writing – review & editing. SH: Writing – review & editing. SL: Writing – review & editing. FSc: Writing – review & editing. FSt: Writing – review & editing. MS: Writing – review & editing. AB: Formal analysis, Writing – review & editing. AMei: Formal analysis, Investigation, Writing – review & editing. AA: Formal analysis, Investigation, Methodology, Writing – review & editing. PM: Formal analysis, Writing – review & editing, Conceptualization, Data curation, Funding acquisition, Investigation, Methodology, Project administration, Resources, Supervision, Validation.

## References

[1] Punga AR Maddison P Heckmann JM Guptill JT Evoli A. Epidemiology, diagnostics, and biomarkers of autoimmune neuromuscular junction disorders. Lancet Neurol. (2022) 21:176–88. doi: 10.1016/S1474-4422(21)00297-0, PMID: 35065040

[2] Gilhus NE. Myasthenia gravis. N Engl J Med. (2016) 375:2570–81. doi: 10.1056/NEJMra1602678 28029925

[3] Gilhus NE Skeie GO Romi F Lazaridis K Zisimopoulou P Tzartos S. Myasthenia gravis – autoantibody characteristics and their implications for therapy. Nat Rev Neurol. (2016) 12:259–68. doi: 10.1038/nrneurol.2016.44, PMID: 27103470

[4] Huijbers MG Marx A Plomp JJ Le Panse R Phillips WD. Advances in the understanding of disease mechanisms of autoimmune neuromuscular junction disorders. Lancet Neurol. (2022) 21:163–75. doi: 10.1016/S1474-4422(21)00357-4 35065039

[5] Meriggioli MN Sanders DB. Autoimmune myasthenia gravis: emerging clinical and biological heterogeneity. Lancet Neurol. (2009) 8:475–90. doi: 10.1016/S1474-4422(09)70063-8, PMID: 19375665 PMC2730933

[6] Howard JF. Myasthenia gravis: the role of complement at the neuromuscular junction. Ann N Y Acad Sci. (2017) 1412:113–28. doi: 10.1111/nyas.13522 29266249

[7] Sieb JP. Myasthenia gravis: an update for the clinician. Clin Exp Immunol. (2014) 175:408–18. doi: 10.1111/cei.12217, PMID: 24117026 PMC3927901

[8] Roper J Fleming ME Long B Koyfman A. Myasthenia gravis and crisis: evaluation and management in the emergency department. J Emerg Med. (2017) 53:843–53. doi: 10.1016/j.jemermed.2017.06.009, PMID: 28916122

[9] Gamez J Salvadó M Carmona F de Nadal M Romero L Ruiz D. Intravenous immunoglobulin to prevent myasthenic crisis after thymectomy and other procedures can be omitted in patients with well-controlled myasthenia gravis. Ther Adv Neurol Disord. (2019) 12:1756286419864497. doi: 10.1177/1756286419864497, PMID: 31360225 PMC6640060

[10] Neumann B Angstwurm K Mergenthaler P Kohler S Schönenberger S Bösel J. Myasthenic crisis demanding mechanical ventilation: a multicenter analysis of 250 cases. Neurology. (2020) 94:e299–313. doi: 10.1212/WNL.0000000000008688 31801833

[11] Liu Z Yao S Zhou Q Deng Z Zou J Feng H. Predictors of extubation outcomes following myasthenic crisis. J Int Med Res. (2016) 44:1524–33. doi: 10.1177/0300060516669893, PMID: 27856933 PMC5536745

[12] Alshekhlee A Miles JD Katirji B Preston DC Kaminski HJ. Incidence and mortality rates of myasthenia gravis and myasthenic crisis in us hospitals. Neurology. (2009) 72:1548–54. doi: 10.1212/WNL.0b013e3181a41211, PMID: 19414721

[13] Mergenthaler P Stetefeld HR Dohmen C Kohler S Schönenberger S Bösel J. Seronegative myasthenic crisis: a multicenter analysis. J Neurol. (2022) 269:3904–11. doi: 10.1007/s00415-022-11023-z, PMID: 35389099 PMC8988104

[14] O'Riordan JI Miller DH Mottershead JP Hirsch NP Howard RS. The management and outcome of patients with myasthenia gravis treated acutely in a neurological intensive care unit. Eur J Neurol. (1998) 5:137–42. doi: 10.1046/j.1468-1331.1998.520137.x, PMID: 10210824

[15] Kalita J Kohat AK Misra UK. Predictors of outcome of myasthenic crisis. Neurol Sci. (2014) 35:1109–14. doi: 10.1007/s10072-014-1659-y 24497206

[16] König N Stetefeld HR Dohmen C Mergenthaler P Kohler S Schönenberger S. Musk-antibodies are associated with worse outcome in myasthenic crisis requiring mechanical ventilation. J Neurol. (2021) 268:4824–33. doi: 10.1007/s00415-021-10603-9, PMID: 33970337 PMC8563593

[17] Angstwurm K Vidal A Stetefeld H Dohmen C Mergenthaler P Kohler S. Early tracheostomy is associated with shorter ventilation time and duration of Icu stay in patients with myasthenic crisis-a multicenter analysis. J Intensive Care Med. (2022) 37:32–40. doi: 10.1177/0885066620967646 33233998

[18] Liu F Wang Q Chen X. Myasthenic crisis treated in a Chinese neurological intensive care unit: clinical features, mortality, outcomes, and predictors of survival. BMC Neurol. (2019) 19:172. doi: 10.1186/s12883-019-1384-5 31324153 PMC6642475

[19] Nelke C Stascheit F Eckert C Pawlitzki M Schroeter CB Huntemann N. Independent risk factors for myasthenic crisis and disease exacerbation in a retrospective cohort of myasthenia gravis patients. J Neuroinflammation. (2022) 19:89. doi: 10.1186/s12974-022-02448-4, PMID: 35413850 PMC9005160

[20] Jiang Z Ning Z Yang L Chen B Tang J Zhang J. The correlation of neutrophil-to-lymphocyte ratio with the presence and short-time curative effect of myasthenia gravis in children: a retrospectively study. Int J Neurosci. (2021) 131:894–901. doi: 10.1080/00207454.2020.1759592 32315556

[21] Yang DH Qian MZ Wei MM Li J Yu MM Lu XM. The correlation of neutrophil-to-lymphocyte ratio with the presence and activity of myasthenia gravis. Oncotarget. (2017) 8:76099–107. doi: 10.18632/oncotarget.18546 29100295 PMC5652689

[22] Hsu CW Chen NC Huang WC Lin HC Tsai WC Huang CC. Hemogram parameters can predict in-hospital mortality of patients with myasthenic crisis. BMC Neurol. (2021) 21:388. doi: 10.1186/s12883-021-02412-4, PMID: 34615473 PMC8493047

[23] Mehnert A Bershan S Kollmus-Heege J Gerischer L Herdick ML Hoffmann S. Identifying patients at risk for myasthenic crisis with hemogram and inflammation-related laboratory parameters – a pilot study. medRxiv. (2023). doi: 10.1101/2023.09.19.23295421 PMC10925644 38469587

[24] R Core Team. R: A language and environment for statistical computing. Vienna, Austria: R Foundation for Statistical Computing (2021).

[25] Therneau TM. A package for survival analysis in R. (2021). Available at: https://CRAN.R-project.org/package=survival.

[26] Therneau TM Grambsch PM. The Cox model In: Dietz K Gail M Krickberg K Samet J Tsiatis A. Eds. Modeling survival data: extending the Cox model. New York, NY: Springer (2000). 39–77.

[27] Højsgaard S Halekoh U Yan J. The R package Geepack for generalized estimating equations. J Stat Softw. (2005) 15:1–11. doi: 10.18637/jss.v015.i02

[28] Gummi RR Kukulka NA Deroche CB Govindarajan R. Factors associated with acute exacerbations of myasthenia gravis. Muscle Nerve. (2019) 60:693–9. doi: 10.1002/mus.26689 31469909

[29] Lotan I Hellmann MA Wilf-Yarkoni A Steiner I. Exacerbation of myasthenia gravis following corticosteroid treatment: what is the evidence? A systematic review. J Neurol. (2021) 268:4573–86. doi: 10.1007/s00415-020-10264-0 33064188

[30] Abuzinadah AR Alanazy MH Butt NS Barohn RJ Dimachkie MM. Exacerbation rate in generalized myasthenia gravis and its predictors. Eur Neurol. (2021) 84:43–8. doi: 10.1159/000512077, PMID: 33321491 PMC7969373

[31] de Meel RH Lipka AF van Zwet EW Niks EH Verschuuren JJ. Prognostic factors for exacerbations and emergency treatments in myasthenia gravis. J Neuroimmunol. (2015) 282:123–5. doi: 10.1016/j.jneuroim.2015.03.018, PMID: 25903739

[32] Wang L Zhang Y He M. Clinical predictors for the prognosis of myasthenia gravis. BMC Neurol. (2017) 17:77. doi: 10.1186/s12883-017-0857-7, PMID: 28420327 PMC5395963

[33] Kato T Kawaguchi K Fukui T Nakamura S Hakiri S Nakatochi M. Risk factors for the exacerbation of myasthenic symptoms after surgical therapy for myasthenia gravis and thymoma. Semin Thorac Cardiovasc Surg. (2020) 32:378–85. doi: 10.1053/j.semtcvs.2019.09.002 31518701

[34] Akaishi T Motomura M Shiraishi H Yoshimura S Abe M Ishii T. Preoperative risks of post-operative myasthenic crisis (Pomc): a meta-analysis. J Neurol Sci. (2019) 407:116530. doi: 10.1016/j.jns.2019.116530, PMID: 31654832

[35] Kanai T Uzawa A Sato Y Suzuki S Kawaguchi N Himuro K. A clinical predictive score for postoperative myasthenic crisis. Ann Neurol. (2017) 82:841–9. doi: 10.1002/ana.25087, PMID: 29083502

[36] Lv Z Zhong H Huan X Song J Yan C Zhou L. Predictive score for in-hospital mortality of myasthenic crisis: a retrospective Chinese cohort study. Eur Neurol. (2019) 81:287–93. doi: 10.1159/000503961 31661691

[37] Gilhus NE Romi F Hong Y Skeie GO. Myasthenia gravis and infectious disease. J Neurol. (2018) 265:1251–8. doi: 10.1007/s00415-018-8751-9 29372387

[38] Sy RW Chawantanpipat C Richmond DR Kritharides L. Thrombocytopenia and mortality in infective endocarditis. J Am Coll Cardiol. (2008) 51:1824–5. doi: 10.1016/j.jacc.2008.01.034 18452791

[39] Hu SL Said FR Epstein D Lokeshwari M. The impact of anemia on renal recovery and survival in acute kidney injury. Clin Nephrol. (2013) 79:221–8. doi: 10.5414/CN107471, PMID: 23253902

[40] Menon V Lessard D Yarzebski J Furman MI Gore JM Goldberg RJ. Leukocytosis and adverse hospital outcomes after acute myocardial infarction. Am J Cardiol. (2003) 92:368–72. doi: 10.1016/s0002-9149(03)00651-9, PMID: 12914863

[41] Shu DH Ransom TP O'Connell CM Cox JL Kaiser SM Gee SA. Anemia is an independent risk for mortality after acute myocardial infarction in patients with and without diabetes. Cardiovasc Diabetol. (2006) 5:8. doi: 10.1186/1475-2840-5-8, PMID: 16603081 PMC1459852

[42] Miyake K Karasuyama H. Emerging roles of basophils in allergic inflammation. Allergol Int. (2017) 66:382–91. doi: 10.1016/j.alit.2017.04.007 28506528

[43] Stone KD Prussin C Metcalfe DD. Ige, mast cells, basophils, and eosinophils. J Allergy Clin Immunol. (2010) 125:S73–80. doi: 10.1016/j.jaci.2009.11.017, PMID: 20176269 PMC2847274

[44] Bershan S Meisel A Mergenthaler P. Classifying the risk for myasthenic crisis using data-driven explainable machine learning with informative feature design and variance control – a pilot study. medRxiv. (2023). doi: 10.1101/2023.08.19.23294175

